# MAB_2355c Confers Macrolide Resistance in Mycobacterium abscessus by Ribosome Protection

**DOI:** 10.1128/AAC.00330-21

**Published:** 2021-07-16

**Authors:** Qi Guo, Yongjie Zhang, Junsheng Fan, Haonan Zhang, Zhemin Zhang, Bing Li, Haiqing Chu

**Affiliations:** a Department of Respiratory Medicine, Shanghai Pulmonary Hospital, Tongji University School of Medicine, Shanghai, China; b Tongji University School of Medicine, Shanghai, China; c Shanghai Key Laboratory of Tuberculosis, Shanghai Pulmonary Hospital, Tongji University School of Medicine, Shanghai, China

**Keywords:** ribosome, ABC-F protein, MAB_2355c, *Mycobacterium abscessus*, macrolides, resistance

## Abstract

Macrolide resistance is always a concern when treating Mycobacterium abscessus infections. MAB_2355c was identified previously as a possible new factor that confers the intrinsic resistance of 194 clinical M. abscessus isolates to clarithromycin. Herein, the potential mechanism by which MAB_2355c exerts macrolide resistance was explored by bioinformatics analysis, *MAB_2355c* cloning and protein purification, ATP hydrolysis assay, gene knockout and complementation, antibiotic sensitivity, and transcription-translation assays. MAB_2355c is a putative ATP-binding cassette F (ABC-F) family protein. Purified MAB_2355c protein exhibits ATP hydrolysis activity, which can be inhibited by ribosome-targeting antibiotics. *MAB_2355c* mRNA expression is upregulated more significantly after exposure to macrolides than after exposure to other ribosome-targeting antibiotics. *MAB_2355c* deleted strains showed increased sensitivity to macrolides, which was reduced by *MAB_2355c* complementation. Finally, MAB_2355c rescued the transcription and translation activities affected by macrolides *in vitro*. These findings suggest that MAB_2355c confers the resistance of M. abscessus to macrolides by ribosome protection, thus complementing other known resistance mechanisms.

## INTRODUCTION

Mycobacterium abscessus is an opportunistic human pathogen that is ubiquitous in the environment and is capable of causing a wide range of diseases in immunocompetent, as well as immunocompromised, hosts (1–4). The rate of M. abscessus detection has increased in recent years (1, 5–7); indeed, M. abscessus is now described as a “neglected global threat” (1). Although rare and somewhat controversial, human-to-human transmission of virulent clones observed among cystic fibrosis patients makes the problem even more disconcerting (8–10).

M. abscessus is intrinsically resistance to most antimicrobial agents (11, 12). Among the few available antibacterial drugs, macrolides are recommended as the treatment of choice due to their anti-M. abscessus activity, as well as their inherent anti-inflammatory and immunomodulatory effects (6, 7, 13). Treatment success rates are poor, however, particularly in the case of macrolide resistance strains (14–16). Modification in the macrolide binding target, e.g., a 2270/2271 point mutation in the 23S rRNA (*rrl*) gene or thymine located at position 28 in the full-length *erm*(41) gene [*erm*(41)full-T28 sequevar], is the primary mechanism that results in decreased macrolide sensitivity (17, 18). These known mechanisms, however, fail to account fully for the resistance to macrolides exhibited by M. abscessus (19, 20).

The results of a previous study involving 194 clinical M. abscessus isolates suggest that the efflux pump MAB_2355c might be a new factor which contributes to intrinsic clarithromycin resistance (21). Herein, the potential mechanism was explored. The results indicate that MAB_2355c can be characterized as a putative ATP-binding cassette F (ABC-F) family protein, the first reported in M. abscessus. MAB_2355c exhibits ATP hydrolysis activity and can restore microbial transcription and translation, which are otherwise inhibited by macrolides. This is the first study to suggest that MAB_2355c facilitates macrolide resistance by ribosome protection. Moreover, it extends our understanding of the factors that affect the resistance of M. abscessus to macrolides and suggests novel approaches to treating macrolide-resistant infections.

## RESULTS

### MAB_2355c is an ATP-binding cassette F family protein.

**FIG 1:**
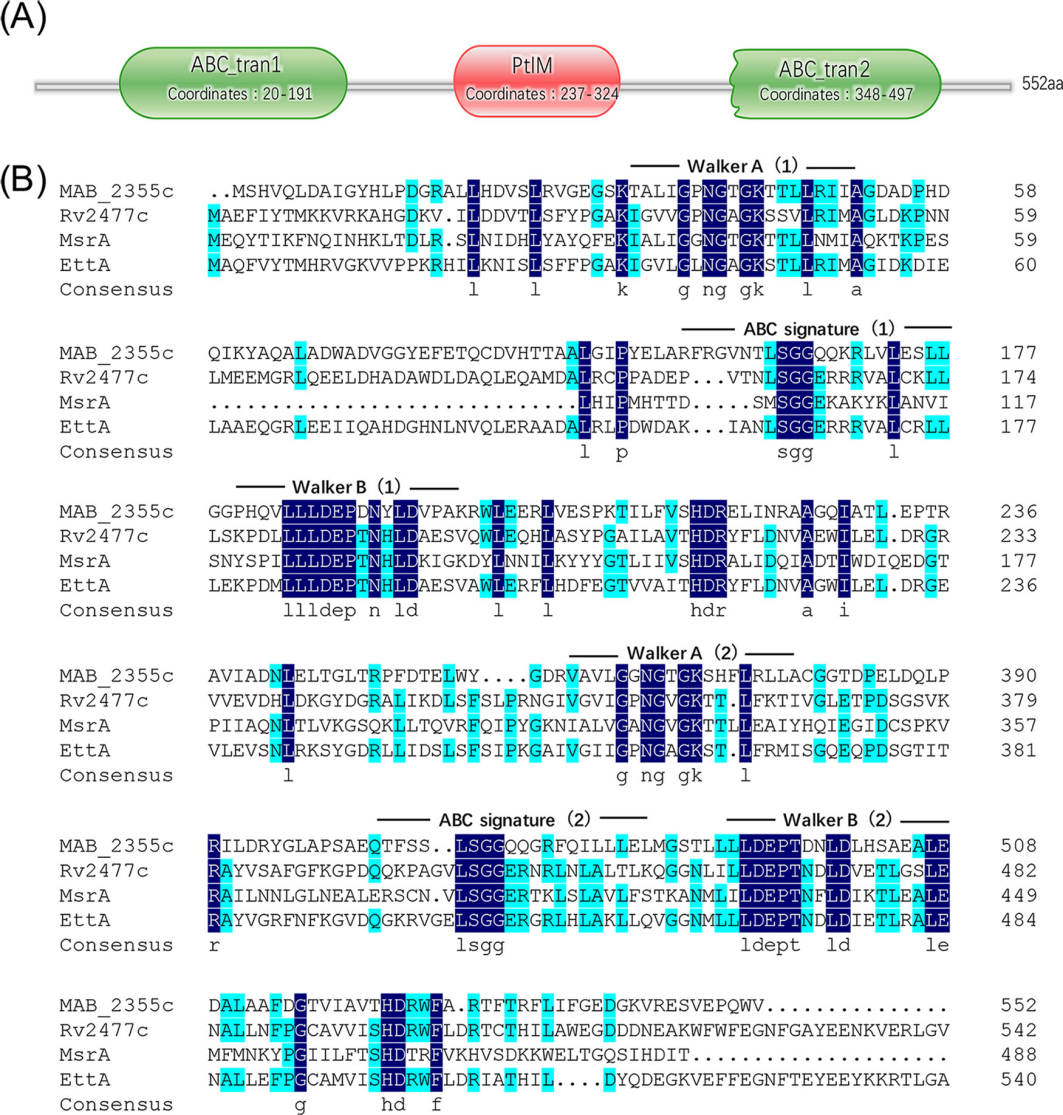
MAB_2355c exhibits similarities to ABC-F subfamily proteins. (A) Schematic diagram of the conserved domains of MAB_2355c predicted by the Pfam database. ABC_tran (PF00005) and ABC_tran_Xtn (PtIM, PF12848) were found in MAB_2355c. (B) The amino acid sequences of the ABC-F subfamily proteins were aligned and shaded using DNAMAN software. Dark blue represents 100% homology, and light blue represents 75% homology. The amino acid sequences used for alignment include MAB_2355c (GenBank accession numbers CAM62436), Rv2477c (CCP45271), MsrA (ADM29228), and EttA (P0A9W3). The annotation above the MAB_2355c sequence indicates the following conserved motifs: Walker A, ABC signature, Walker B.

The protein encoded by *MAB_2355c* is annotated in NCBI as a “putative ABC transporter ATP-binding protein.” Bioinformatic analyses indicate that MAB_2355c contains no transmembrane domains and displays homology to ATP-binding cassette F (ABC-F) proteins reported previously (19). MAB_2355c exhibits the characteristics of typical ABC-F family proteins, i.e., two tandem ATP-binding domains (also called nucleotide binding domains, NBD) connected by a specific number of residue linkers (Fig. 1A) (22, 23). The Pfam database indicates that the ABC domain, which includes typical Walker A and Walker B motifs, belongs to the conserved domain group ABC_tran (PF00005), a typical feature of ABC transporters and ATP hydrolysis proteins (Fig. 1B). The linker in MAB_2355c protein is also identified as the ABC_tran_Xtn (PF12848) domain. The ABC_tran_Xtn domain can form an α-helical hairpin that interacts directly with tRNA in the P site and binds to the peptidyl tRNA in ribosomes, also known as the P site tRNA interaction motif (PtIM) (Fig. 1A) (23).

### MAB_2355c exhibits ATP hydrolysis activity, which can be inhibited by antibiotics.

**FIG 2:**
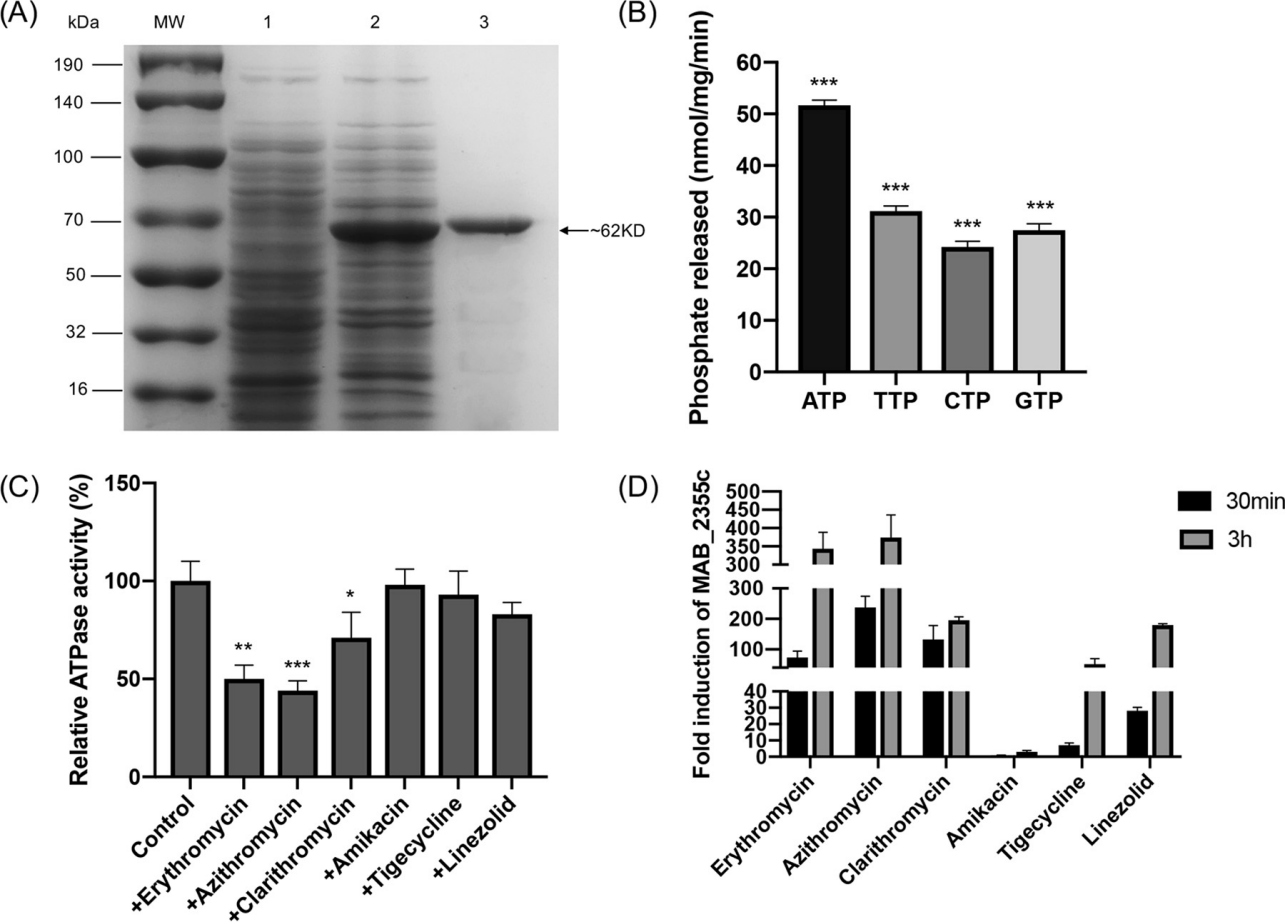
Characterization of purified MAB_2355c ATPase activity and changes in *MAB_2355c* expression after exposure to ribosome-targeting antibiotics. (A) Coomassie-stained SDS-PAGE confirmed the accuracy of MAB_2355c induction and purification. MW, molecular weight marker. Lane 1, before IPTG induction; lane 2, after IPTG induction; and lane 3, purified MAB_2355c protein. (B) The MAB_2355c protein exhibits ATP, TTP, CTP, and GTP hydrolase activity. (C) MAB_2355c protein (1 μM) was preincubated with 1 mM antibiotic for 15 min at room temperature prior to the addition of ATP. ATP hydrolysis was quantified after deducting the background (solvent rather than antibiotic added). Data in panels C and D represent the mean ± standard deviation (SD), and statistical significance was analyzed by one-tailed *t* test versus buffer controls. *, *P* < 0.05; **, *P* < 0.01; ***, *P* < 0.001. (D) Fold *MAB_2355c* transcript induction in wild-type M. abscessus ATCC 19977 after 30 min and 3 h exposure to 0.5 MIC ribosome-targeting antibiotics. The results are expressed as fold increased expression compared to unexposed samples. Data represent the mean ± SD, sigA was used as an endogenous reference gene. All experiments were repeated independently three times.

MAB_2355c was expressed first as an N-terminal histidine-tagged fusion protein in BL21 cells and then purified by Ni-NTA prepacked column chromatography. The recombinant protein (∼62 kDa) was strongly expressed in heterologous host cells (Fig. 2A). The ability of purified MAB_2355c to hydrolyze ATP was determined by phorbol myristate acetate (PMA)-malachite green spectrophotometry. The results showed that the ATP hydrolysis activity of MAB_2355c was ∼51.7 nmol/mg/min. MAB_2355c was also capable of hydrolyzing GTP, TTP, and CTP but to a lesser extent than ATP (Fig. 2B).

Reportedly, antibiotics can inhibit the ATPase activity of ABC-F proteins (24). To determine whether antibiotics affected MAB_2355c ATPase activity, purified MAB_2355c was preincubated with different concentrations of antibiotics known to bind ribosomal subunits, and then the ATPase activity was assayed. As shown, the ATPase activity of MAB_2355c was inhibited by antibiotics; the inhibitory effect of macrolides was the most obvious (Fig. 2C).

### Macrolide exposure induces *MAB_2355c* expression.

The expression of *MAB_2355c* was assessed after M. abscessus was exposed to sublethal concentrations of ribosomal-targeting antibiotics (Fig. 2D). Macrolides were strong inducers of *MAB_2355c* expression. Expression increased significantly after 30 min incubation. Less expression was observed after 30 min exposure to other antibiotics. Macrolides also induced more *MAB_2355c* expression than did other antibiotics after prolonged (3 h) exposure.

### *MAB_2355c* deletion results in increased macrolide sensitivity.

**FIG 3:**
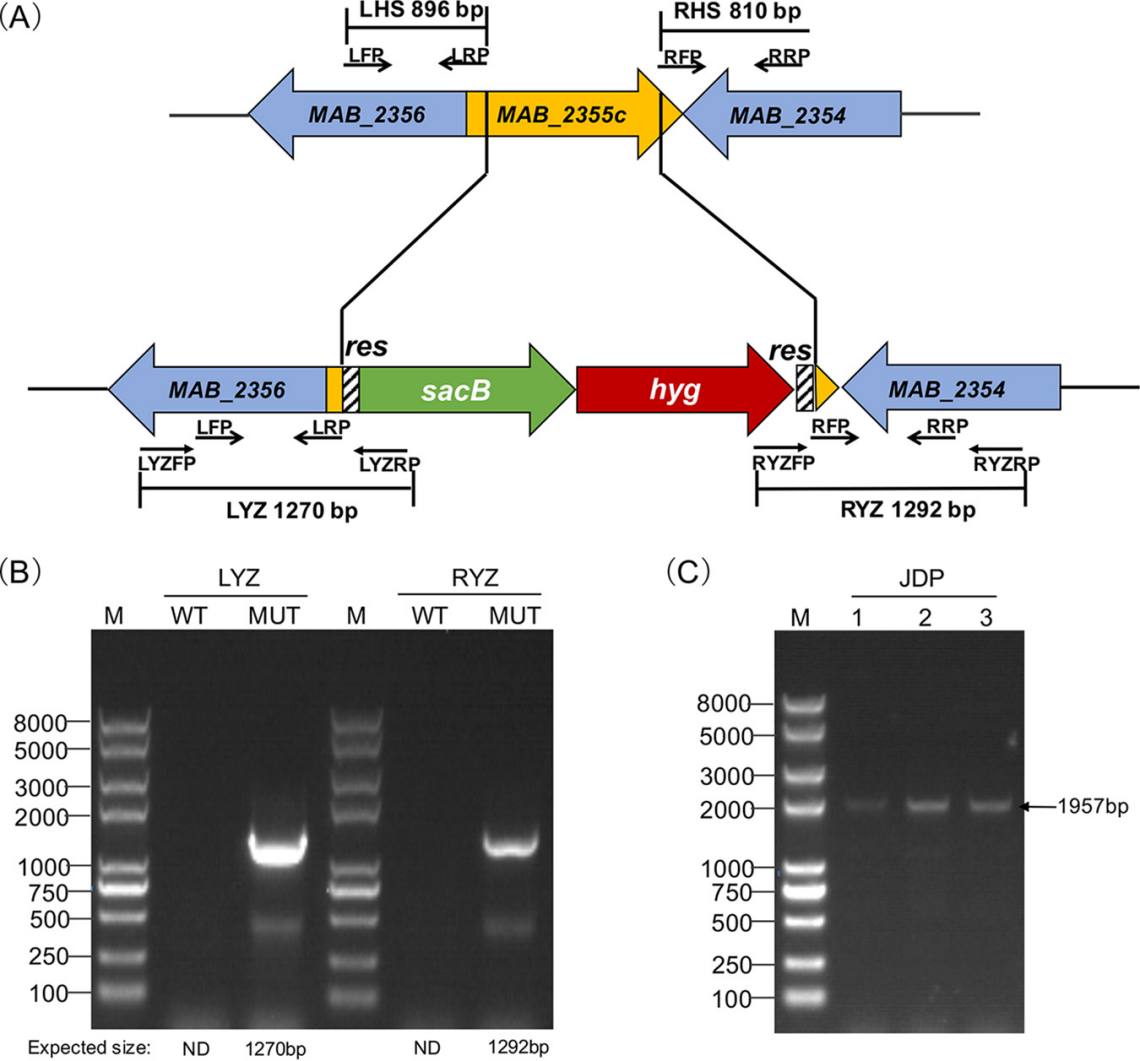
Construction of knockout and complementation strains. (A) Schematic representation of the *MAB_2355c* deletion created using phage recombineering. (B) Using LYZFP/LYZRP and RYZFP/RYZRP primer pairs, 1,270 bp LYZ and 1,292 bp RYZ were amplified, respectively, confirming *MAB_2355c* deletion; the knockout strain (MUT) was used as a template. No targeted DNA fragment was amplified using the wild-type strain (WT) as a template. (C) Three complementation clones were randomly selected (numbers 1 to 3), and the JDP DNA fragment was amplified using the JDLP and JDRP primers and confirmed by sequencing.

**FIG 4:**
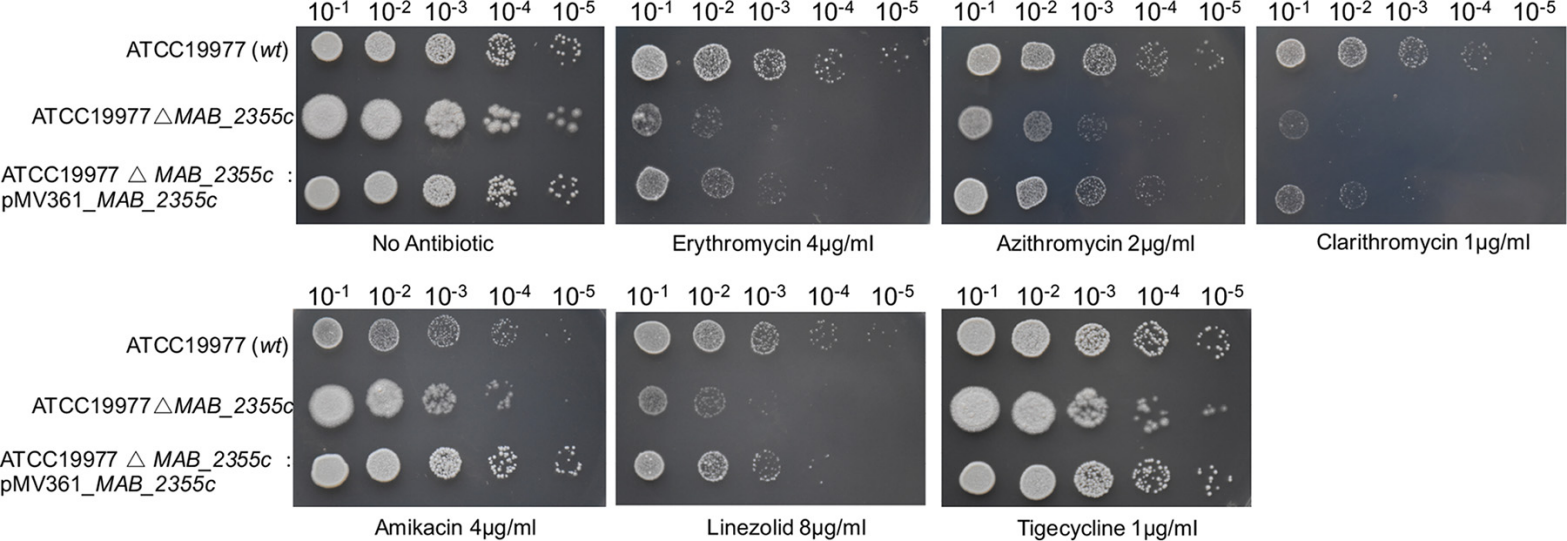
*MAB_2355c* deletion renders M. abscessus sensitive to macrolides. Ten-fold dilutions of ATCC 19977 wild-type, ATCC 19977Δ*MAB_2355c* mutant, and ATCC 19977Δ*MAB_2355c*:pMV361_*MAB_2355c* complementation strains were spotted onto Middlebrook 7H10 agar plates containing the indicated antibiotic concentrations. *MAB_2355c* deletion rendered M. abscessus ATCC 19977 more sensitive than the wild-type parental strain to erythromycin, azithromycin, clarithromycin, and linezolid. An integrated, constitutively expressed copy of *MAB_2355c* in the complementation strain partially restored antibiotic resistance.

To explore further the potential role of *MAB_2355c* in macrolide resistance, knockout and complementation M. abscessus strains were constructed (Fig. 3). ATCC 19977 wild-type, ATCC 19977Δ*MAB_2355c* mutant, and ATCC 19977Δ*MAB_2355c*: pMV361_*MAB_2355c* complementation strains were tested for susceptibility to ribosome-targeting antibiotics by spotting 10-fold serial dilutions of each strain on Middlebrook 7H10 agar containing antibiotic at the concentration indicated in Fig. 4. *MAB_2355c* deletion led to increased sensitivity to macrolides; sensitivity was reversed in the complementation strain. The MICs of antibiotics for these same three strains were also determined in liquid media (Table S3), and a consistent result was found.

### MAB_2355c rescues macrolide-affected translation by ribosome protection.

**FIG 5:**
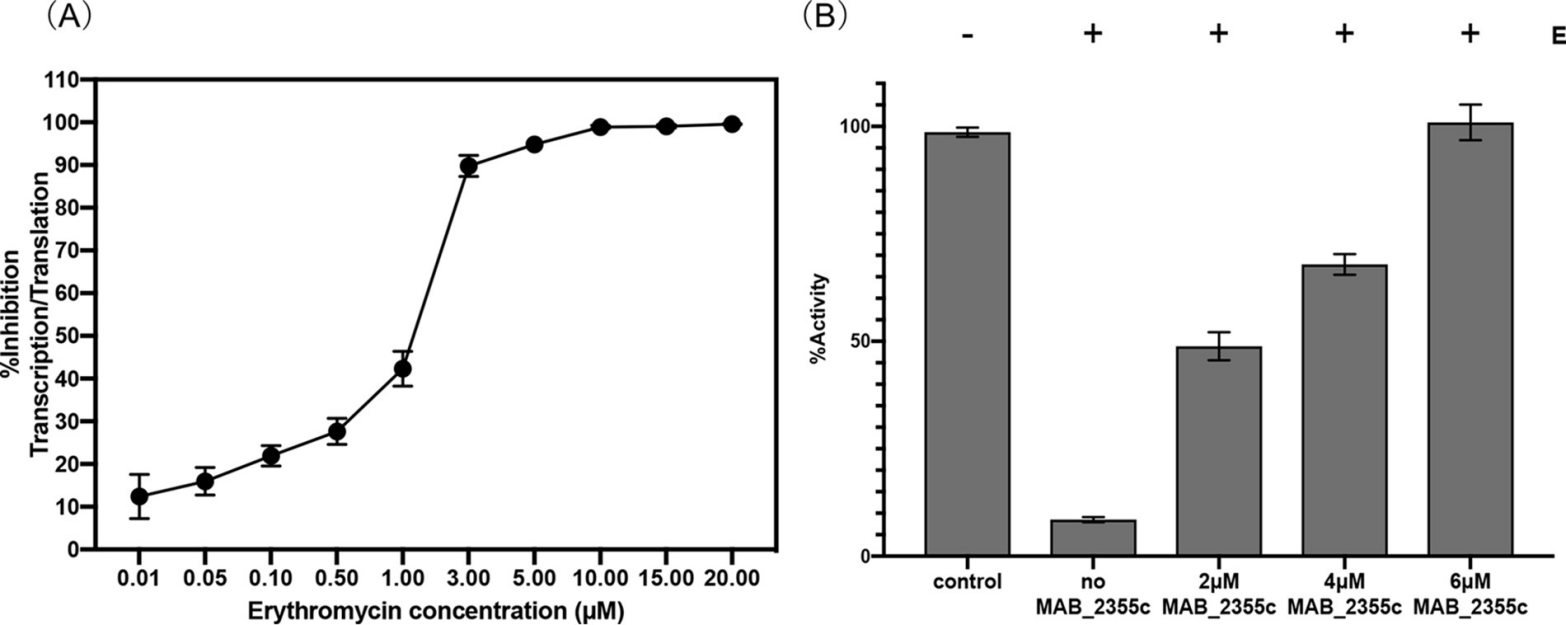
MAB_2355c rescues *in vitro* transcription-translation from erythromycin inhibition in a dose-dependent manner. (A) Erythromycin inhibitory activity profile in an *in vitro* coupled transcription-translation luciferase assay. (B) Transcription-translation activity in the absence of erythromycin and MAB_2355c (column 1) or the presence of 3 μM erythromycin (E) and increasing concentrations of MAB_2355c protein (columns 2 to 5). Results are the means ± SD of data from three independent experiments.

Previous studies suggested that ribosome protection was the mechanism by which ABC-F proteins conferred macrolide resistance (20, 21, 25). The ability of MAB_2355c to protect the translation apparatus of M. abscessus from macrolide-mediated inhibition was tested directly. Erythromycin and purified recombinant MAB_2355c were added to the commercial Escherichia coli S30 *in vitro* coupled transcription-translation assay system. The dose-response inhibition profile of erythromycin in the transcription-translation assay is shown in Fig. 5A. Erythromycin (3 μM) inhibited the *in vitro* transcription-translation assay ∼90%, and the addition of purified MAB_2355c reversed erythromycin-inhibited translation in a dose-dependent fashion (Fig. 5B). These results support ribosome protection as the mechanism by which MAB_2355c confers macrolide resistance.

## DISCUSSION

Macrolides remain the core drugs for treating M. abscessus infections; macrolide-resistant cases have particularly unsatisfactory outcomes (14). The results of a previous study involving 194 clinical M. abscessus isolates indicated that MAB_2355c might confer intrinsic resistance to clarithromycin (21). The present study is the first to report that MAB_2355c in M. abscessus is an antibiotic resistance (ARE), ABC-F subfamily protein. It can hydrolyze ATP without participating directly in transmembrane transport and performs a ribosome protection function that contributes to macrolide resistance (23, 24). Notably, ABC-F genes are found in numerous antibiotic-resistant clinical pathogen and plasmids that confer multidrug resistance (26, 27). As such, ABC-F-mediated target protection may be an important factor contributing to antibiotic resistance in clinical settings.

MAB_2355c exhibits structural features typical of the ABC-F protein family: tandem ABC domains separated by a linker region that contains the P-position tRNA interaction motif. Most ABC superfamily proteins are transmembrane transporters that can mediate the import and export of a wide variety of drugs, nutrients, and even polymers. ABC-F subfamily members are the exception. Subfamily members frequently contain a pair of NBDs without an associated transmembrane domain and are capable of modulating ribosomal mRNA translation in multiple ways. A recent bioinformatics study identified the candidate subfamilies of ABC-F and refined the classification (27). The results showed that ABC-F in bacteria can be divided into ∼30 subfamilies, each with an average of four ABC-Fs per bacterial genome, with considerable variation between subfamilies. Reports describing the ABC-F family in Mycobacterium are limited; to date, the only ABC-F protein reported is Rv2477c in M. tuberculosis (28). Although both *MAB_2355c* and *Rv2477c* are found in Mycobacterium, they do not exhibit a high degree of homology.

The ABC-F subfamilies have well-characterized roles in intracellular processes other than transport, including DNA repair, replication, and translation regulation (23, 29). Recent studies further extended the non-transport-related functions of the ABC-F subfamily by demonstrating that these proteins can mediate antibiotic resistance, specifically to compounds (e.g., ketolides, lincosamides, macrolides, oxazolidinones, phenicols, pleuromutilins, and group A and B streptogramins) that target the 50S ribosomal subunit (27, 30). Previously, we reported that MAB_2355c conferred the intrinsic resistance of clinical M. abscessus isolates to clarithromycin (21). Hurst-Hess et al. reported that *MAB_2355c* was the first and most frequently induced gene in M. abscessus following exposure to 0.5 MIC ribosome-targeting antibiotic (31). *MAB_2355c* expression was significantly reduced following deletion of *whiB7*, a key transcriptional regulator associated with antibiotic resistance. The fact that MAB_2355c exhibits the typical structural features of the ABC-F protein family suggests that it may be capable of inhibiting ribosome-targeting antibiotics. In the present study, the sensitivity of M. abscessus to macrolides (i.e., erythromycin, azithromycin, and clarithromycin) increased after *MAB_2355c* was knocked out. Conversely, the sensitivity was reduced by *MAB_2355c* complementation, confirming that *MAB_2355c* functions as an antibiotic resistance ABC-F subfamily protein. To explore other potential physiologic functions of MAB_2355c, the growth rates of wild-type, knockout, and complementation strains in 7H9 liquid medium were assessed and compared; no difference was observed (data were not shown). Interestingly, though, the morphology of colonies growing on agar plates changed from smooth to rough after *MAB_2355c* deletion; *MAB_2355c* complementation of the deleted strain restored smooth colony formation (Fig. S1). This implies that MAB_2355c may also be involved in membrane and lipid biosynthesis, a function that needs to be investigated in greater detail.

Circumstantial but compelling evidence suggests that ABC-F proteins, such as the Vga/Lsa/Sal-type, provide antibiotic resistance by a mechanism involving ribosomal protection (26, 28, 32). Recent studies, undertaken to explore the mechanism by which ABC-F proteins promote the separation of antibiotics from ribosomes, visualized the structure of MsrE and VmlR binding to ribosomes by cryo-electron microscopy (30, 32). These ABC-F proteins bind the E site of ribosomes with their domain linkers inserted into the peptidyl transferase center (PTC) or nascent peptide exit tunnel (NPET), thus preventing disassociation of tRNA from the ribosome P binding site. Therefore, antibiotic resistance ABC-F proteins appear to bind physically to the ribosome, resulting in the combined effects of a structural shift and a change in ribosome conformation in PTC and NPET, which facilitate antibiotic release. In the current study, MAB_2355c reversed the inhibition of transcription and translation by macrolides *in vitro*, providing additional evidence for an antibiotic resistance mechanism based upon MAB_2355c-mediated drug replacement. More direct evidence is needed, however, focusing on MAB_2355c-ribosome structure interaction.

In conclusion, MAB_2355c is the first reported antibiotic resistance ABC-F family protein in M. abscessus. MAB_2355c exhibits ATP hydrolysis activity and contributes to macrolide resistance by ribosome protection. As such, MAB_2355c complements markers like *rrl* and *erm*(41) that predict macrolide susceptibility and help formulate antibacterial strategies.

## MATERIALS AND METHODS

### Bacteria and media.

M. abscessus ATCC 19977 and Mycobacterium smegmatis mc^2^155 were grown at 37°C in Middlebrook 7H9 medium (BD-Difco) supplemented with 10% oleic acid-albumin-dextrose-catalase (OADC) and 0.05% Tween 20 or on Middlebrook 7H10 agar plates supplemented with 0.5% glycerol. E. coli DH5α and BL21 (Vazyme) were grown at 37°C in Luria broth (LB) or on LB agar plates. Antibacterial drugs were added as indicated.

### Bioinformatics analysis.

The amino acid sequence of *MAB_2355c* and other sequences used for BLAST analysis were obtained from the National Center for Biotechnology Information (Bethesda, MD, USA). Multiple sequences were aligned and similar amino acids in the aligned sequences were shaded using DNAMAN software. The online TMHMM (TMHMM server v.2.0) website was used to predict the protein transmembrane domain. The conserved domains of MAB_2355c were identified from the Pfam database (http://pfam.xfam.org/search/sequence).

### Cloning, induced expression, and purification of recombinant MAB_2355c protein.

All primer sequences and plasmid information used in this study are listed in Tables S1 and S2. The *MAB_2355c* sequence was amplified from M. abscessus ATCC 19977 genomic DNA by PCR using specific primers (*MAB_2355c* DBFP/*MAB_2355c* DBRP). The amplified product was cloned into a linearized pET28a plasmid vector using ClonExpress Ultra one step cloning kit (Vazyme). The recombinant plasmid was verified by sequencing and then transformed into BL21 cells for protein expression.

BL21 cells transformed with the pET28a-*MAB_2355c* recombinant plasmid were grown in LB medium until 0.6 to 0.8 optical density at 600 nm (OD_600_). Then, 0.5 mM isopropyl β-d-thiogalactoside was added and the culture was shaken for 16 h at 22°C to induce recombinant protein expression. Protein expression was confirmed by sodium dodecyl sulfate-polyacrylamide gel electrophoresis and Coomassie blue staining.

Cells from the induced culture were lysed by ultrasonication in the presence of 0.2 mM phenylmethylsulfonyl fluoride on ice. MAB_2355c protein was purified from the lysate at 4°C using Ni-NTA prepacked chromatographic columns (Sangon Biotech, Shanghai, China). The column-bound protein was washed with buffer (50 mM Tris-HCl, 500 mM NaCl, 20 mM imidazole [pH 8.0]) and then eluted with buffer consisting of 50 mM Tris-HCl, 500 mM NaCl, 250 mM imidazole (pH 8.0). The purified protein was concentrated and buffer-exchanged with 50 mM Tris-HCl (pH 8), 300 mM NaCl, and 10% glycerol using 30-kDa cutoff filters (Millipore) and then snap-frozen in liquid nitrogen and stored at –80°C.

### ATP hydrolysis assays.

The ATP hydrolase activity of purified MAB_2355c was determined by PMA-Malachite green spectrophotometry. The reaction buffer was prepared according to the description provided with the ultra-trace total ATPase detection kit (Nanjing Jiancheng Institute of Biological Engineering). To determine whether MAB_2355c was capable of hydrolyzing other nucleotide triphosphates, CTP, GTP, or TTP was added to the reaction buffer in lieu of ATP. Malachite green was added after 10 min incubation at 37°C and the absorbance at 636 nm was measured. ATP hydrolysis by purified protein was estimated from the amount of phosphate released. To investigate the effects of antibiotics on the ATPase activity of MAB_2355c, 1 μM purified MAB_2355c protein was incubated for 15 min at room temperature with 1 mM antibiotic prior to adding ATP (25, 28). For the control, the same volume of solvent, rather than antibiotic, was added to the reaction mixture. Phosphate contamination was avoided throughout the enzymatic reaction. Experiments were repeated independently three times.

### RNA extraction and qPCR.

Wild-type M. abscessus ATCC 19977 was exposed to 0.5 MIC ribosome-targeting antibiotics for either 30 min or 3 h. Total RNA was extracted and quantitative real-time PCR (qRT-PCR) was performed according to the protocol described previously (21). PCR primer pairs used for amplification were *sigA* _RT_F/R (*sigA* is an endogenous reference gene) and *MAB_2355c*_RT_F/R.

### Construction of knockout and complementation strains.

The ATCC 19977Δ*MAB_2355c* knockout strain was constructed according to methods described by Jain et al. (33). The LFP/LRP and RFP/RRP primer sets were used to amplify the left and right flanking sequences of *MAB_2355c* from the wild-type ATCC 19977 genome by PCR. The p0004s plasmid carrying the sacB-hyg cassette was used to construct the allelic exchange substrate (AES). The p0004s plasmid and the left and right flanking sequences were digested with Van91I restriction endonuclease and then ligated with T4 DNA ligase. E. coli DH5α was transformed with the ligation mixture, and the clone was confirmed by sequencing. The mycobacteriophage vector phAE159 and p0004s-AES positive plasmids were extracted and digested with PacI. The two linearized plasmid fragments were ligated with T4 DNA ligase. E. coli HB101 was transformed with the reaction mixture using a packaging kit (EPICENTRE Biotechnologies). The phAE159-AES positive plasmid was screened by growth on a hygromycin-resistant (150 μg/ml) LB agar plate and identified by PacI restriction enzyme digestion. M. smegmatis mc^2^155 competent cells were electroporated (2.5 kV, 1,000 Ω, 25 μF) with the resultant phAE159-AES plasmid to obtain phage. M. abscessus that grew logarithmically to 0.6 to 0.8 OD_600_ was washed with MP buffer (50 mM Tris-HCl, 150 mM NaCl, 10 mM MgSO4, 2 mM CaCl2 [pH 7.5 to 7.8]) and incubated with phage lysate (titer of ∼1,010) at 37°C for 2 days. The culture was centrifuged, the bacteria in the pellet were suspended and spread onto Hyg-resistant agar plates, and the plates were incubated at 37°C for 3 days. Mutant clones were verified using LYZFP/LYZRP and RYZFP/RYZRP primers.

To create the ATCC 19977Δ*MAB_2355c*:pMV361_*MAB_2355c* complementation strain, the *MAB_2355c* DNA sequence in the M. abscessus ATCC 19977 genome was amplified using the following primer set: *MAB_2355c* FP/*MAB_2355c* RP. The amplified sequence was recovered, recombined with linearized pMV361 plasmid digested with EcoRI and HindIII, and then transformed into DH5α to select and amplify the recombinant plasmid. M. abscessus ATCC 19977Δ*MAB_2355c* was electrotransformed with the recombinant pMV361-*MAB_2355c* plasmid and then spread onto resistant 7H10 agar plates for selection. Mutant clones were checked by PCR.

### Antimicrobial drug sensitivity assay.

The wild-type, knockout, and complementation strains were grown to 0.6 to 0.7 OD_600_. The susceptibility of the cells to ribosome-targeting antibiotics was tested by first spotting 10-fold serial dilution on Middlebrook 7H10 agar plates containing a range of each drug: 0.5 to 16.0 μg/ml erythromycin, 0.5 to 8.0 μg/ml azithromycin, 0.125 to 4.0 μg/ml clarithromycin, 1.0 to 16.0 μg/ml amikacin, 1.0 to 32.0 μg/ml linezolid, and 0.25 to 16.0 μg/ml tigecycline. The plates were incubated at 37°C until colonies grew. Simultaneously, the sensitivity of the three stains to the same antibiotics was determined by the micro broth dilution method according to the Clinical and Laboratory Standards Institute (CLSI)-M24-A2 guidelines. Mycobacterium peregrinum (ATCC 700686; American Type Culture Collection, Manassas, VA, USA) and Staphylococcus aureus ATCC 29213 served as control reference strains. The results were performed in duplicate.

### *In vitro* transcription-translation assay.

E. coli S30 extract system for circular DNA was used (Promega Biotechnology Company, Madison, WI, USA). Briefly, 5 μl of S30 extract was added to 20 μl reaction mixture consisting of 0.1 mM amino acids, 10 μl S30 premix, 1 μg of pBESTluc DNA template, and specified concentrations of erythromycin and purified MAB_2355c protein as indicated. Samples were incubated for 1 h at 37°C followed by 5 min inactivation on ice. The luciferase assay reagent (Promega) was added, and transcription-translation was quantified by monitoring luciferase activity and luminescence using Varioskan Flash (Thermo Fisher Scientific).

### Statistical analysis.

The experiments were performed in triplicate. Differences between groups were analyzed by using Prism 8 and one-tailed *t* test. ***, *P* < 0.001, **, *P* < 0.01, *, *P* < 0.05, means ± standard error of the mean from at least three biological replicates.
