# Supplementary material for: MAB_2355c Confers Macrolide Resistance in Mycobacterium abscessus by Ribosome Protection
Source: Antimicrob Agents Chemother. 2021 Jul 16;65(8):e00330-21. doi: 10.1128/AAC.00330-21 (PMC8373217; doi:10.1128/AAC.00330-21)

Supplemental Table 1. Bacteria and plasmids used in this study

Strain or plasmid	Description	Source
Strains		
E. coli		
DH5α	For routine cloning procedures	Purchased from Vazyme
BL21	For expression of MAB_2355c	Purchased from Vazyme
HB101	For plasmid phAE159-AES construction	Purchased from Vazyme
M. abscessus		
ATCC19977	Standard strain of *M. abscessus*	Laboratory preservation
ATCC19977△MAB_2355c	Derivative of *M. abscessus* that lacks *MAB_2355c* gene	This study
ATCC19977△MAB_2355c: pMV_MAB_2355	ATCC19977△MAB_2355c with wild-type *MAB_2355c*	This study
M. smegmatis mc^{2}155	For phage construction	Laboratory preservation
Plasmids		
pET28a	For expression of MAB_2355c with His tag in *E. coli*, with kan resistanc	Laboratory preservation
phAE159	Phagemid with Amp resistance	Purchased from Gene Optimal Biotech
p0004s	For the construction of homology arms, with Hyg B resistance	Purchased from Gene Optimal Biotech
pMV361	For the construction of complemented plasmid, with kan resistance	Purchased from Gene Optimal Biotech

Supplemental Table 2. List of primers used in this study

Primer name	Primer sequence（5'-3'）
MAB_2355c DBFP	CAGCAAATGGGTCGCGGATCCATGAGCCATGTGCAGCTCG
MAB_2355c DBRP	ACGGAGCTCGAATTCGGATCCTTATACCCATTGGGGTTCAACG
sigA_RT_F	AGCGTGAGCTGCTACAGGAC
sigA_RT_R	TGGATTTCCAGCACCTTCTC
MAB_2355c_RT_F	CTGGCCAGCTCATACGGAAT
MAB_2355c_RT_R	ACTCATGGAGTGCGACAGTG
LFP	TTTTTTTTCCATAAATTGGAGCTGGCCCATATCGGCACGT
LRP	TTTTTTTTCCATTTCTTGGTATCAGGGCGGTCTTGCTGCC
RRP	TTTTTTTTCCATCTTTTGGCCAACGACTACGTGCAGTTGCA
LYZFP	GTGGCAAGTGTGTCAGGCAAGTT
LYZRP	GTGGACCTCGACGACCCTAG
RYZFP	TGGATCTCTCCGGCTTCACC
RYZRP	CGGAACAGACGTCGTCGCAGAAT
MAB_2355c HBFP	TCCAGCTGCAGAATTCATGAGCCATGTGCAGCTCGATG
MAB_2355c HBRP	CGACATCGATAAGCTTTTATACCCATTGGGGTTCAACGG
JDFP	GTGGCAGCGAGGACAACTTG
JDRP	CCCGACGTCAGGTGGCTAG

Supplemental Table 3. Antimicrobial susceptibility of wild-type, *MAB_2355c* knockout and complementary *M. abscessus* ATCC 19977 strains

**Antibiotics**	**MICs (μg/ml)**		
	**ATCC19977 *WT***	**ATCC19977 △*MAB_2355c***	**ATCC19977 △*MAB_2355c*: pMV361_*MAB_***
Erythromycin	1	0.25	0.5
Azithromycin	2	0.5	2
Clarithromycin	0.5	0.125	0.5
Amikacin	8	8	8
Imipenem	16	16	16
Meropenem	32	32	32
Linezolid	8	8	8
Cefoxitin	16	16	16
Moxifloxacin	4	4	4
Ciprofloxacin	8	8	8
Minocycline	8	8	8
Tigecycline	0.5	0.5	0.5
Tobramycin	8	8	8

Supplemental Figure 1. Colonies of the ATCC 19977：*MAB_2355c* mutant undergo morphological transformation. The colonial morphologies of ATCC19977 wild-type, ATCC19977Δ*MAB_2355c* mutant and ATCC19977Δ*MAB_2355*:pMV361_*MAB_2355c* complementation strains grown on 7h10 agar plates for 5 days are shown.

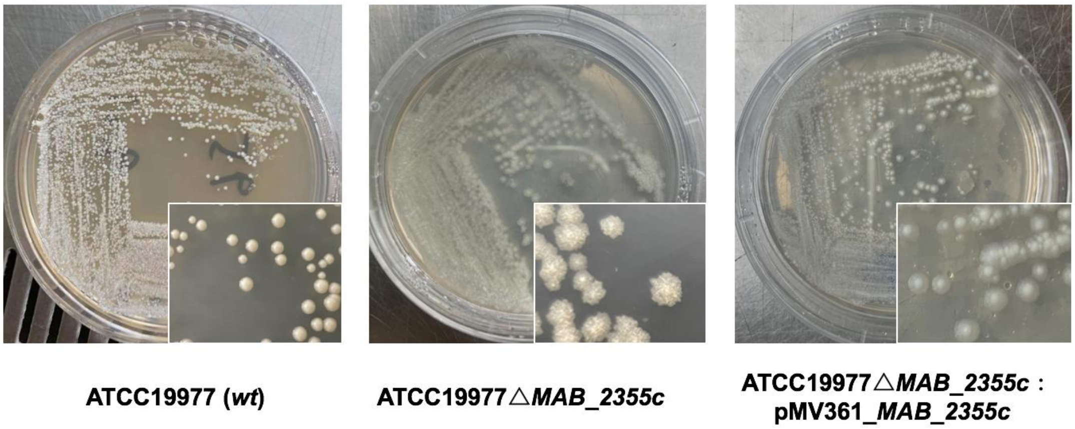

Supplement: Supplemental file 1 — Supplemental tables and figure. Download AAC.00330-21-s0001.pdf, PDF file, 0.3 MB [file aac.00330-21-s0001.pdf]
